# RSP5 Positively Regulates the Osteogenic Differentiation of Mesenchymal Stem Cells by Activating the K63-Linked Ubiquitination of Akt

**DOI:** 10.1155/2020/7073805

**Published:** 2020-04-06

**Authors:** Changxiang Liang, Guoyan Liang, Xiaoqing Zheng, Yongxiong Huang, Shuaihao Huang, Dong Yin

**Affiliations:** Department of Spinal Surgery, Guangdong Provincial People's Hospital, Guangzhou, China 510080

## Abstract

Mesenchymal stem cells (MSCs) are multipotent stem cells that have a strong osteogenic differentiation capacity. However, the molecular mechanism underlying the osteogenic differentiation of MSCs remains largely unknown and thus hinders further development of MSC-based cell therapies for bone repair in the clinic. RSP5, also called NEDD4L (NEDD4-like E3 ubiquitin protein ligase), belongs to the HECT (homologous to E6-AP carboxyl terminus) domain-containing E3 ligase family. Nevertheless, although many studies have been conducted to elucidate the role of RSP5 in various biological processes, its effect on osteogenesis remains elusive. In this study, we demonstrated that the expression of RSP5 was elevated during the osteogenesis of MSCs and positively regulated the osteogenic capacity of MSCs by inducing K63-linked polyubiquitination and activation of the Akt pathway. Taken together, our findings suggest that RSP5 may be a promising target to improve therapeutic efficiency by using MSCs for bone regeneration and repair.

## 1. Introduction

Mesenchymal stem cells (MSCs) are multipotent stem cells that have a strong osteogenic differentiation capacity [1, 2]. Due to their strong potential in osteogenic differentiation, MSCs are considered to be the most promising cell types used in tissue engineering technology for bone regeneration and repair [1, 2]. However, the molecular mechanism that modulates the osteogenic differentiation of MSCs remains largely unknown and thus hinders further development of MSC-based cell therapies for bone repair in the clinic. Therefore, for the use of MSCs in therapeutic applications, further exploration of the mechanism underlying osteogenic differentiation is needed.

RSP5, also called NEDD4L (NEDD4-like E3 ubiquitin protein ligase), belongs to the HECT (homologous to E6-AP carboxyl terminus) domain-containing E3 ligase family [3, 4]. Previous studies have demonstrated that the HECT domain-containing family plays an important role in bone formation. For example, Smurf1/2 negatively regulates the osteogenic differentiation of MSCs by degrading Smad1 and Runx2, while NEDDL4 positively regulates the osteogenesis of MSCs by activating the pSmad2 and pERK1/2 pathways [5–8]. Nevertheless, although many studies have been conducted to elucidate the role of RSP5 in various biological processes, its effect on bone formation remains elusive [9, 10].

The serine/threonine protein kinase Akt participates in many aspects of biological functions, such as cell proliferation, metabolism, cell cycle, and metastasis [11]. Multiple studies have confirmed that the Akt signaling pathway plays an important role in osteogenesis [12, 13]. The activation of Akt is regulated through Akt phosphorylation at Thr308 and Ser473 [11]. However, recent studies focused on Akt phosphorylation have indicated that ubiquitination and deubiquitination of Akt are also on-off switches for Akt activity [11]. For instance, necrosis factor receptor-associated factor 6 (TRAF6) and Skp2-Skp-cullin-F-box-containing (SCF) regulate Akt activation as an E3 ligase through Lys63(K63)-linked polyubiquitination of Akt in insulin-like growth factor 1 (IGF-1) and ErbB receptor signaling, respectively [11, 14]. Although multiple studies have explored Akt phosphorylation and activation during osteogenesis, the concrete mechanism of Lys63(K63)-linked polyubiquitination of Akt during osteogenesis remains largely unknown.

In this study, we focused on exploring the effect of RSP5 on the osteogenic potential of MSCs and further clarified the concrete mechanism. We aimed to determine whether RSP5 can act as a checkpoint in cell fate to regulate the osteogenic differentiation of MSCs.

## 2. Materials and Methods

### 2.1. Ethics Statement

This study conforms to the Declaration of Helsinki and was approved by the Ethics Committee of Guangdong Provincial People's Hospital. Nine healthy donors between the ages of 20 and 30 years old were recruited in the study. Before the study, all healthy donors were informed of the clinical requirements and possible risks of all operations and signed informed consent was obtained.

### 2.2. Cell Isolation and Expansion

Bone marrow aspirations were performed by a skilled doctor. MSCs in the bone marrow samples were isolated immediately using a density gradient centrifugation method. Briefly, the bone marrow samples were transferred to low-glucose Dulbecco's modified Eagle's medium (DMEM, Gibco) containing 10% fetal calf serum (FBS, Gibco) to a total volume of 10 ml and then added to 10 ml Percoll (Pharmacia Biotech) at a density of 1.073 g/ml. The mononuclear cells were isolated by gradient centrifugation at 900 g for 30 min. The isolated mononuclear cells were washed with phosphate-buffered saline (PBS) and then seeded in a culture flask with low-glucose DMEM supplemented with 10% FBS. The cells were cultured at 37°C and 5% CO_2_, and the culture medium was replaced every 2 days. MSCs were passaged when the culture reached 90% confluency. MSCs at passage 2 were used for the experiments.

### 2.3. Surface Marker Identification

MSCs were digested using 0.25% trypsin containing 0.53 mM EDTA, and the reaction was terminated with FBS. After the MSCs were washed by PBS three times, they were incubated with antibodies against CD14, CD29, CD44, CD45, CD105, and HLA-DR (Miltenyi Biotec) for 30 min according to the protocols. MSCs were washed with PBS three times, and the positive rate of the surface markers was detected by a BD Influx cell sorter (BD Biosciences).

### 2.4. Trilineage Differentiation Potential Assay

MSCs were cultured and induced to undergo trilineage differentiation: osteogenic differentiation, chondrogenic differentiation, and adipogenic differentiation. For osteogenic differentiation, MSCs were seeded in 12-well plates at a density of 1.5 × 10^4^ cells/cm^2^ and cultured in osteogenic differentiation medium containing DMEM with 10% FBS, 100 IU/ml penicillin, 100 IU/ml streptomycin, 0.1 *μ*M dexamethasone, 10 mM *β*-glycerol phosphate, and 50 *μ*M ascorbic acid (Sigma). The medium was replaced every three days, and the osteogenic differentiation potential was determined by Alizarin red S (ARS) and alkaline phosphatase (ALP) staining and quantification. For adipogenic differentiation, MSCs were seeded as described above and cultured in adipogenic differentiation medium containing DMEM with 10% FBS, 1 *μ*M dexamethasone, 10 *μ*g/ml insulin (Sigma), 0.5 mM 3-isobutyl-1-methylxanthine (Sigma), and 0.2 mM indomethacin (Sigma). After 3 days of induction, the medium was replaced every three days, and the adipogenic differentiation potential was determined by Oil red O staining. For chondrogenic differentiation, 2.5 × 10^5^ MSCs were centrifuged at 600 g for 5 min in 15 ml polypropylene conical tubes to form pellets as previously described [15]. The pellets were cultured in chondrogenic differentiation medium containing 100 IU/ml penicillin, 100 IU/ml streptomycin, 1% ITS-Premix (Corning), 50 M ascorbic acid (Sigma), 1 mM sodium pyruvate (Sigma), 0.1 M dexamethasone, and 10 ng/ml transforming growth factor-*β*3 (R&D). The medium was replaced every three days. The pellets were subjected to Alcian blue staining to determine the chondrogenic differentiation potential.

### 2.5. Cell Proliferation Assay

The proliferation rate of MSCs was determined via the Cell Counting Kit-8 (CCK-8, Dojindo) assay according to the manufacturer's protocol. Medium lacking MSCs was used as a negative control.

### 2.6. ARS Assays

For ARS staining, MSCs were fixed and stained with 1% ARS for 10 min. The cells were washed using PBS 3 times. Images of stained cells were taken under a microscope. For ARS quantification, the cells were cultured with 10% cetylpyridinium chloride monohydrate (CPC, Sigma-Aldrich) for 30 min with gentle shaking. The absorbance of the extracted supernatant was measured at 562 nm by a microplate reader (Thermo Fisher).

### 2.7. ALP Assays

For ALP activity, MSCs were lysed in RIPA lysis buffer (Thermo Fisher) containing protease inhibitors and phosphatase inhibitors (Roche). The lysate was centrifuged at 12,000 rpm at 4°C for 30 min. The supernatant was extracted, and the ALP activity in the protein supernatant was detected using ALP activity kits (Nanjing Jiancheng Biotech) according to the manufacturer's protocol. For ALP staining, MSCs were fixed in a citrate-acetone-formaldehyde fixative and then treated with an alkaline dye (Sigma-Aldrich) for 15 min in the dark. Images of stained cells were taken under a microscope.

### 2.8. Oil Red O Staining

MSCs were fixed with 4% paraformaldehyde for 15 min. After the MSCs were washed with PBS three times, they were stained with Oil red O working solution for 15 min. Images of the stained cells were taken under a microscope.

### 2.9. Alcian Blue Staining

The cell pellets were fixed with 4% paraformaldehyde and then embedded in paraffin to slice into sections. The sections were stained using Alcian blue solution for 30 min and washed with PBS three times. Images of the stained cells were taken under a microscope.

### 2.10. Western Blotting

Total protein of the MSCs was extracted as described above [16]. The protein concentration in the supernatant was measured using a BCA assay kit (Sigma-Aldrich). The proteins were separated by sodium dodecyl sulfate-polyacrylamide gel electrophoresis, followed by transfer to a polyvinylidene fluoride membrane (Millipore). The membrane was incubated with primary antibodies against GAPDH, RSP5, Smad1, phosphorylated Smad1/5/9, total catenin, nonphosphorylated catenin, Akt, and phosphorylated Akt (1 : 1000, Abcam) overnight. After the membrane was washed by TBST buffer, it was incubated with horseradish peroxidase (HRP)-conjugated secondary antibody (1 : 3000, Abcam) for 1 h. Specific antibody-antigen complexes were detected using the Immobilon Western Chemiluminescent HRP Substrate (Millipore).

### 2.11. Knockdown and Overexpression Lentivirus Infection

For knockdown lentivirus construction, four siRNAs for RSP5 were designed, and the most effective siRNA was chosen to construct the knockdown lentiviruses. The sequence for RSP5 was 5′-ACGTCTCGCATTTGAGCAGGG-3′, and the sequence for the negative control was 5′-TTCTCCGAACGTGTCACGTTTC-3′. For overexpression lentiviruses, the complete nucleotide sequences of RSP5 were constructed. Both knockdown and overexpression lentiviruses were generated by the GenePharma Company. Lentiviruses (10^9^ TU/ml) with 5 *μ*g/ml polybrene were incubated with MSCs for 24 h at an MOI of 50. Related experiments were performed as described on day 14 of osteogenic differentiation.

### 2.12. Quantitative Real-Time PCR

Total RNA of MSCs was extracted using an RNA-Quick Purification Kit (Yishan Biotech) according to the protocols. The extracted RNA was synthesized into cDNA using PrimeScript™ RT reagent kits (TaKaRa). Quantitative real-time PCR was performed on a LightCycler® 480 PCR system (Roche) using SYBR® Premix Ex Taq™ kits (TaKaRa). The relative expression levels of each gene were analyzed using the 2^−^^ΔΔ^^Ct^ method and normalized to *β*-actin expression. The sequences of the forward and reverse primers for each gene are shown below (Table 1).

### 2.13. Akt Pathway Blocking

AZD5363 (Selleck) was added at a concentration of 5 *μ*M as MSCs underwent osteogenic differentiation. Related experiments were performed on day 10 of induction.

### 2.14. Coimmunoprecipitation Assay

MSC proteins were extracted as described above [17]. The protein supernatant was incubated with antibodies against RSP5, Akt, or the IgG (Abcam) control at 4°C overnight. The protein-G agarose beads were then added to the mixture and incubated at 4°C for 3 h. The beads were collected and washed five times, followed by resuspension, and boiling in buffer containing 50 mM Tris, 2% sodium dodecyl sulfonate (SDS), 10% glycerol, 10 mM dithiothreitol (DTT), and 0.2% bromophenol blue. All the samples were detected using western blotting assays as described above [17].

### 2.15. Plasmid Construction and Transfection

Expression plasmids including pcDNA3.1(+)-HA-UB, pcDNA3.1(+)-HA-K63-UB, pcDNA3.1(+)-HA-K48-UB, pcDNA3.1(+)-Myc-RSP5, and pcDNA3.1(+)-Flag-Akt were all purchased from Obio Technology Corp, Ltd. For plasmid transfection, 293 T cells were seeded in 6-well plates. A total of 2 *μ*g/well of each plasmid with 5 *μ*l of Lipo3000 and 5 *μ*l of P3000 were added and incubated with 293 T cells for 2 days. The proteins of the 293 T cells were extracted after treatment with 10 *μ*M cycloheximide, and then, IP and western blotting assays were performed as described above. The primary antibodies against Flag-Tag, Myc-Tag, and HA-Tag were all from Abcam.

### 2.16. Statistical Analysis

All data are expressed as the mean ± standard deviation (SD). *T* tests and one-way analysis of variance followed by the Bonferroni test and Pearson correlation test were performed for statistical analyses using SPSS (SPSS, Inc.). *P* values less than 0.05 were considered statistically significant. All the results were determined based on three separate experiments containing triplicate samples.

## 3. Results

### 3.1. Phenotype and Trilineage Differentiation Capacity of MSCs

**Figure 1:**
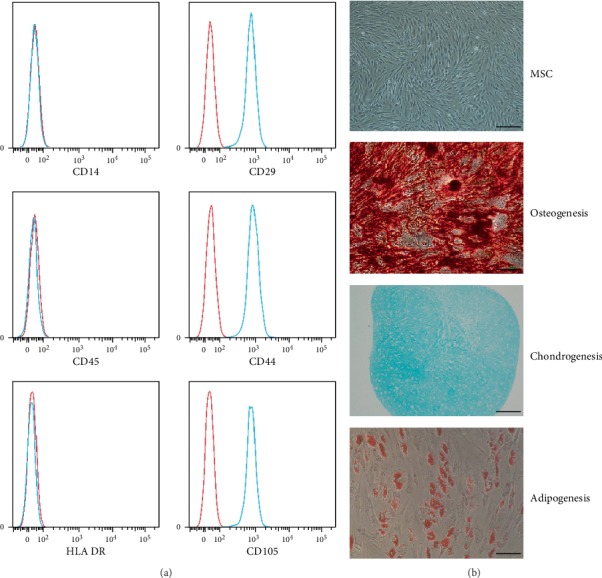
Phenotype and trilineage differentiation capacity of MSCs. (a) MSCs were positive for CD29, CD44, and CD105 but negative for CD14, CD45, and HLA-DR. (b) The MSCs were spindle-shaped before induction. The MSCs could be induced to undergo osteogenic differentiation, chondrogenic differentiation, and adipogenic differentiation. Scale bar = 100 *μ*m. *n* = 3 independent experiments with 3 different MSC lines.

To identify MSCs and determine their purity, we detected the cell phenotypes by flow cytometry. The results showed that the MSCs were positive for CD29, CD44, and CD105 but negative for CD14, CD45, and HLA-DR, which was consistent with the typical phenotype observed in previous reports (Figure 1(a)) [18]. In addition, the MSCs were spindle-shaped and fibroblast-like cells. These cells could be induced to undergo osteogenic differentiation, chondrogenic differentiation, and adipogenic differentiation in specific inducing medium (Figure 1(b)). These results showed that MSCs met the identification criterion of the International Society of Cell Therapy and were of high purity [18].

### 3.2. RSP5 Expression in MSCs during Osteogenic Differentiation

**Figure 2:**
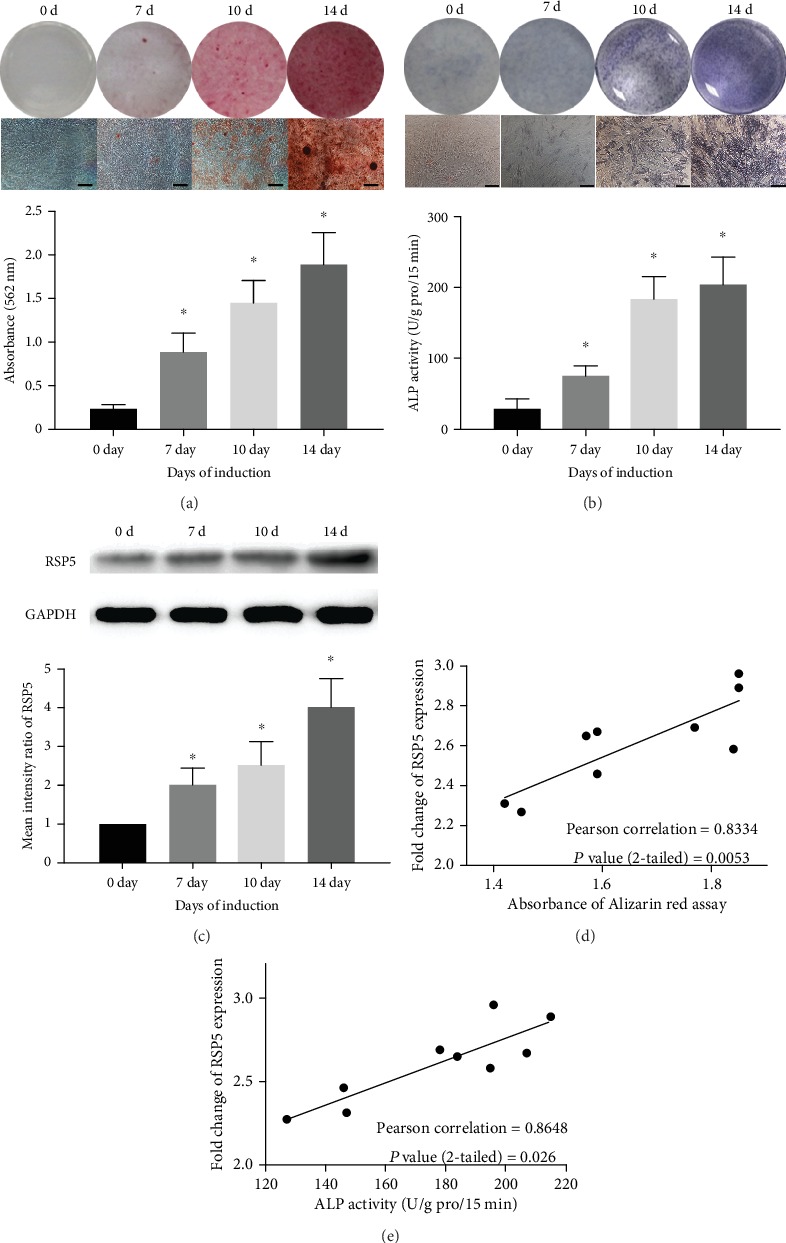
RSP5 expression in MSCs during osteogenic differentiation. (a) The staining of ARS became darker from day 0 to 14 of induction. The quantification of ARS staining was also increased from day 0 to 14 of induction. Scale bar = 100 *μ*m. (b) The staining of ALP was also darker from day 0 to 14 of induction. The ALP activity increased from day 0 to 14 of induction. Scale bar = 100 *μ*m. (c) The expression of RSP5 in at the protein level gradually increased during MSC osteogenic differentiation. (d) The RSP5 expression level was positively related to ARS quantification of MSCs on day 10 of induction. (e) The RSP5 expression level was positively related to the ALP activities of MSCs on day 10 of induction. ^∗^ indicates *P* < 0.05. Scale bar = 100 *μ*m. *n* = 3 independent experiments with 3 different MSC lines.

To detect RSP5 expression during MSC osteogenic differentiation, we first induced MSCs to undergo osteogenic differentiation, and the osteogenic differentiation capacity was determined by ARS and ALP assays at different time points. As shown in Figure 2(a), the number of calcium nodules stained by ARS increased from day 0 to 14 of induction. The quantification of ARS staining gradually rose after induction, showing that MSCs underwent osteogenic differentiation. The ALP assay showed consistent results (Figure 2(b)). In addition, RSP5 expression increased with MSCs undergoing osteogenic differentiation (Figure 2(c)). Moreover, the RSP5 expression level of different MSCs was positively correlated to their osteogenic differentiation capacity as determined by ARS and ALP assays, indicating the strong relationship between RSP5 expression and osteogenic differentiation in MSCs (Figures 2(d) and 2(e)).

### 3.3. Inhibiting RSP5 Expression Decreased the Osteogenic Differentiation Capacity of MSCs

**Figure 3:**
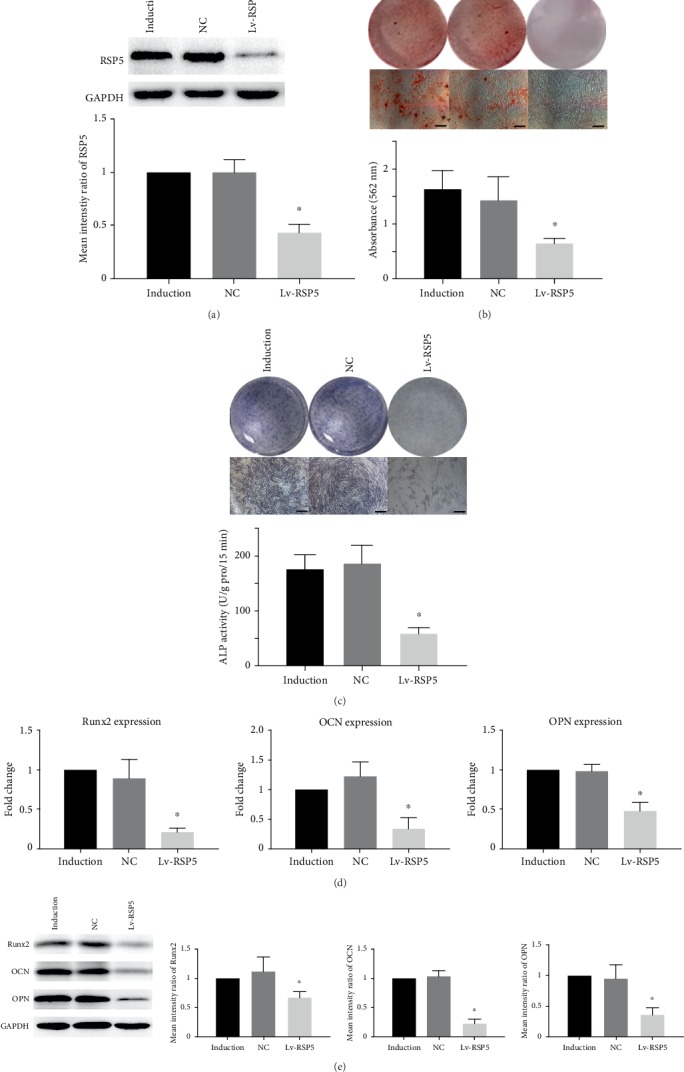
Inhibiting RSP5 expression decreased the osteogenic differentiation capacity of MSCs. (a) Lv-RSP5 significantly inhibited RSP5 expression in MSCs. (b) The ARS staining and quantification of the Lv-RSP5 group were weakened compared to those of the NC group. Scale bar = 100 *μ*m. (c) The ALP staining and activity of the Lv-RSP5 group decreased compared to those of the NC group. Scale bar = 100 *μ*m. (d) Runx2, OCN, and OPN expressions at the gene level were lower in the Lv-RSP5 group than the NC group. (e) The Runx2, OCN, and OPN protein expression levels were also lower in the Lv-RSP5 group than the NC group. ^∗^ indicates *P* < 0.05. Scale bar = 100 *μ*m. *n* = 3 independent experiments with 3 different MSC lines. The induction group indicates MSCs undergoing osteogenic differentiation without other treatment. The NC group indicates MSCs transfected with control lentiviruses undergoing osteogenic differentiation. The Lv-RSP5 group indicates MSCs transfected with lentiviruses encoding an shRNA for RSP5 undergoing osteogenic differentiation.

To clarify the role of RSP5 in the osteogenic differentiation of MSCs, we constructed lentiviruses encoding an shRNA for RSP5 (Lv-RSP5), and the following experiments to explore the role of RSP5 in osteogenic differentiation were conducted after 14 days of osteogenic induction. The inhibitory effect was confirmed by the western blotting results (Figure 3(a)). Knocking down RSP5 did not affect the growth curve of MSCs during osteogenic induction (Supplemental Figure 1A). After the inhibition of RSP5 expression, not only ARS staining and quantification but also ALP activity and staining of MSCs were significantly reduced compared to those in both the induction group and the control lentivirus group (Figures 3(b) and 3(c)). *Runx2*, *OCN*, and *OPN* are critical markers of the osteogenesis of MSCs [1]. The expression levels of all these markers in the Lv-RSP5 group were also decreased at the gene level (Figure 3(d)). Moreover, consistent results of the expression of these markers at the protein level were confirmed by western blotting assays (Figure 3(e)). These results indicated that RSP5 promoted the osteogenic differentiation capacity of MSCs, and decreasing RSP5 expression inhibited MSC osteogenesis.

### 3.4. RSP5 Overexpression Accelerated MSC Osteogenic Differentiation

**Figure 4:**
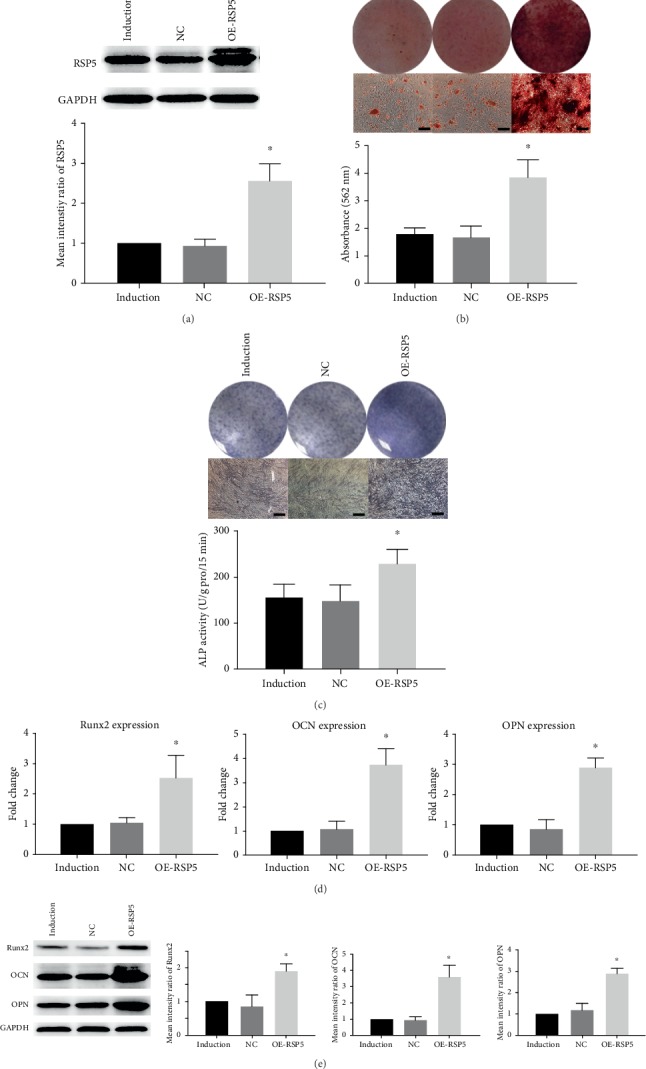
RSP5 overexpression accelerated MSC osteogenic differentiation. (a) OE-RSP5 significantly increased RSP5 expression in MSCs. (b) The ARS staining and quantification of the OE-RSP5 group were higher than those of the NC group. Scale bar = 100 *μ*m. (c) The ALP staining and activity of the OE-RSP5 group were also higher than those of the NC group. Scale bar = 100 *μ*m. (d) Runx2, OCN, and OPN expressions at the gene level increased in the OE-RSP5 group. (e) The Runx2, OCN, and OPN protein expression levels also increased in the OE-RSP5 group. ^∗^ indicates *P* < 0.05. Scale bar = 100 *μ*m. *n* = 3 independent experiments with 3 different MSC lines. The induction group indicates MSCs undergoing osteogenic differentiation without other treatment. The NC group indicates MSCs transfected with control lentiviruses undergoing osteogenic differentiation. The OE-RSP5 group indicates MSCs transfected with lentiviruses overexpressing RSP5 undergoing osteogenic differentiation.

To further confirm the effect of RSP5 on the osteogenic differentiation of MSCs, we then constructed another lentivirus that overexpressed RSP5 (OE-RSP5). After the transfection with OE-RSP5, the RSP5 expression of MSCs was almost 2.5-fold higher than that of the control group (Figure 4(a)). Overexpressing RSP5 did not affect the growth curve of the MSCs during osteogenic induction (Supplemental Figure 1A). In addition, ARS and ALP staining and quantification were much higher in the OE-RSP5 group than in the induction and control lentivirus groups (Figures 4(b) and 4(c)). Moreover, *Runx2*, *OCN*, and *OPN* expressions in the MSCs transfected with OE-RSP5 were significantly increased at both the gene and protein levels (Figures 4(d) and 4(e)). These results confirmed that RSP5 accelerated the osteogenic differentiation capacity of the MSCs.

### 3.5. RSP5 Regulated the Osteogenic Differentiation of MSCs through the Akt Signaling Pathway

**Figure 5:**
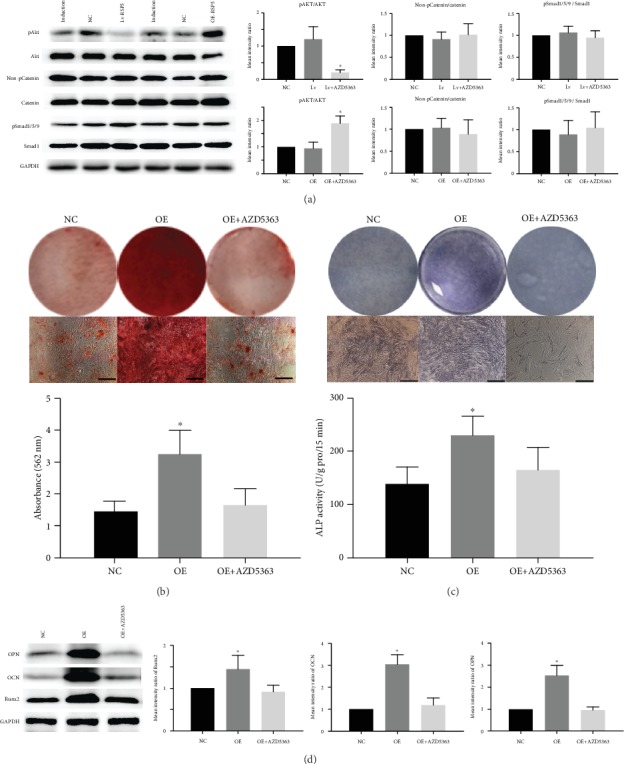
RSP5 regulated the osteogenic differentiation of MSCs through the Akt signaling pathway. (a) The phosphorylated level of Akt was decreased in the Lv-RSP5 group but increased in the OE-RSP5 group. The phosphorylated levels of Smad and catenin were comparable in the Lv-RSP5 group and OE-RSP5 group. (b) OE-RSP5 increased ARS staining and quantification. AZD5363 returned these values to the normal levels in the NC group. Scale bar = 100 *μ*m. (c) OE-RSP5 increased ALP staining and activity. AZD5363 reversed these effects to normal levels as in the NC group. Scale bar = 100 *μ*m. (d) OE-RSP5 increased Runx2, OCN, and OPN expressions. AZD5363 reduced the expression of these markers to the normal levels in the NC group. ^∗^ indicates *P* < 0.05. Scale bar = 100 *μ*m. *n* = 3 independent experiments with 3 different MSC lines. The induction group indicates MSCs undergoing osteogenic differentiation without other treatment. The NC group indicates MSCs transfected with control lentiviruses for Lv-RSP5 or OE-RSP5 undergoing osteogenic differentiation. The OE-RSP5 group indicates MSCs transfected with lentiviruses overexpressing RSP5 undergoing osteogenic differentiation.

We then detected the activation levels of the Akt, catenin, and Smad signaling pathways, which have been reported to be related to the osteogenesis of MSCs. Although the phosphorylation level of Smad1/5/9 or the level of nonphosphorylated catenin remained unchanged in the MSCs transfected with Lv-RSP5 or OE-RSP5, the phosphorylation level of the Akt signaling pathway was significantly decreased in the Lv-RSP5 group but increased in the OE-RSP5 groups (Figure 5(a)), indicating that RSP5 could positively regulate the activation of the Akt signaling pathway. AZD5363 is an inhibitor of the Akt signaling pathway that decreased the growth curve of MSCs under osteogenic induction conditions (Supplemental Figure 1B). Moreover, AZD5363 substantially reduced ARS and ALP staining and quantification in the MSCs transfected with OE-RSP5 (Figures 5(b) and 5(c)). The expression of osteogenic markers, including *Runx2*, *OCN*, and *OPN*, in the MSCs of the OE-RSP5 groups was also reduced to normal levels after treatment with AZD5363 (Figure 5(d)). These results confirmed that RSP5 promoted MSC osteogenic differentiation through the Akt signaling pathway and that blocking the Akt signaling pathway could inhibit these effects of RSP5.

### 3.6. RSP5 Induced the K63-Linked Ubiquitination of Akt

**Figure 6:**
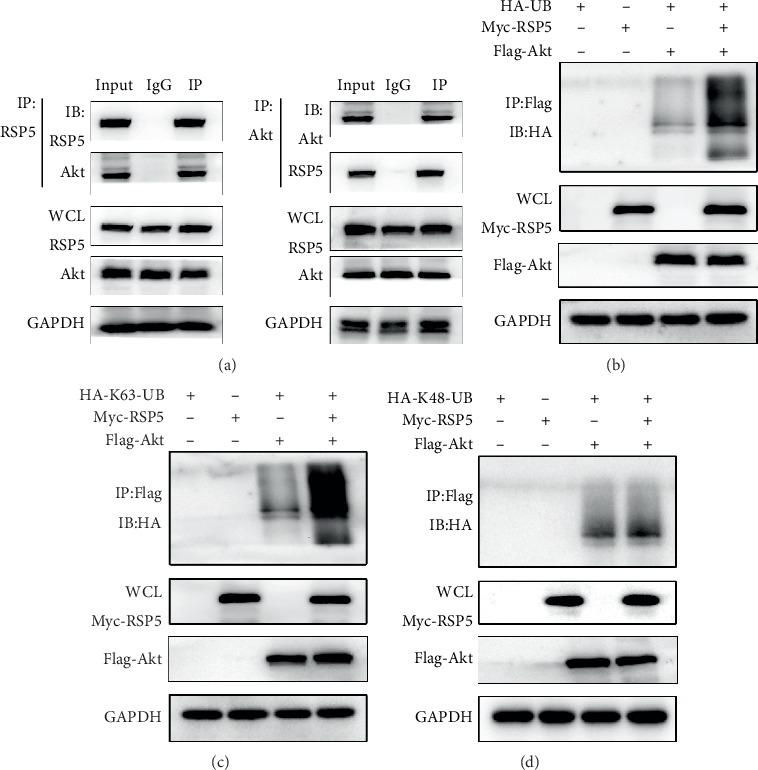
RSP5 induced the K63-linked ubiquitination of Akt. (a) Coimmunoprecipitated mixtures were separated by SDS-PAGE and evaluated by western blots. Endogenous RSP5 and Akt in MSCs interact with each other. (b) Myc-RSP5 significantly induced the ubiquitination of Flag-Akt. (c) Myc-RSP5 significantly induced the K63-linked ubiquitination of Flag-Akt. (d) Myc-RSP5 did not affect the K48-linked ubiquitination of Flag-Akt.

Previous studies have demonstrated that K63-linked ubiquitination of Akt plays an essential role in the phosphorylation and activation of the Akt signaling pathway. We first explored whether RSP5 and Akt interact with each other. Reciprocal Co-IP/western blot assays demonstrated that endogenous RSP5 and Akt interact with each other in MSCs (Figure 6(a)). We then coexpressed HA-Ubiquitin, Myc-RSP5, and Flag-Akt in MSCs and found that Myc-RSP5 significantly induced the ubiquitination of Flag-Akt (Figure 6(b)). Moreover, RSP5 mediated K63-linked ubiquitination of Akt instead of K48-linked ubiquitination (Figures 6(c) and 6(d)). In summary, these data indicated that RSP5 may promote the phosphorylation and activation of the Akt signaling pathway by inducing the K63-linked ubiquitination of Akt.

## 4. Discussion

In this study, we demonstrated that the expression of RSP5 was elevated during the osteogenic differentiation process of MSCs and that RSP5 positively regulated this process by acting as the E3 ubiquitin ligase to mediate the K63-linked ubiquitination and activation of Akt. These findings implied that RSP5 may be a promising target to improve therapeutic efficiency by using MSCs for bone regeneration and repair.

MSCs are multipotent mesenchymal progenitors that can serve as long-term precursors for the differentiation of various cells, including osteoblasts, chondroblasts, and adipocytes [19]. In particular, osteogenic differentiation of MSCs has been widely studied because of its crucial role in multiple physiological and pathological processes [20, 21]. For instance, Tang Y et al. demonstrated that MSCs from patients with systemic lupus erythematosus (SLE) showed a decreased osteogenic capacity and played an important role in the osteoporosis of these patients [20]. Liu X et al. demonstrated that MSCs in patients with ossification of the posterior longitudinal ligament (OPLL) have a high propensity toward osteogenesis and that suppression of osteogenic differentiation in MSCs was effective in treating osteophyte formation in this condition [21]. Moreover, MSCs are considered to be the most promising cell types used in tissue engineering technology for bone regeneration and repair [22]. Thus, studying the regulatory mechanism of osteogenic differentiation of MSCs is helpful for finding new targets and has value in clinical applications.

RSP5, also called NEDD4L, belongs to the HECT domain-containing E3 ligase family [9]. Multiple studies have already confirmed that the expression of RSP5 plays an important role in the development of multiple tissues and organs as well as physiological processes [10, 23, 24]. For instance, Manunta P et al. demonstrated that RSP5 interacted with alpha-adducin to control blood pressure [23], and Kaminska J et al. showed that RSP5 affected the morphology of the actin cytoskeleton in vivo and in vitro [24]. Recently, members of the HECT domain-containing E3 ligase family, including NEDD4, Smurf1, and Smurf2, have been proven to modulate the osteogenic process of MSCs. NEDD4 negatively regulated the osteogenic differentiation of MSCs, and this function was under the control of a long noncoding RNA named SNHG1 [25]. In addition, inhibiting smurf1 expression accelerated the osteogenic differentiation of MSCs and promoted bone regeneration [26]. Moreover, smurf2 was involved in the osteogenic differentiation of MSCs mediated by the NF-*κ*B signaling pathway [27]. However, whether RSP5 plays a role in osteogenesis has never been explored. In this study, we demonstrated that the expression of RSP5 was elevated during the osteogenic induction of MSCs and that manipulating the expression of TRAF4 significantly changed the osteogenic capacity of MSCs. These results indicated that RSP5, similar to other members in its family, may be a potential target to modulate skeletal development by regulating the osteogenic process of MSCs.

To further explore the mechanism of RSP5 in regulating the osteogenic process of MSCs, we first evaluated the activation of several canonical signaling pathways after knocking out or overexpressing RSP5. We demonstrated that after manipulating the expression of RSP5, the activation level of Smad1/5/9 and the catenin signaling pathway remained relatively stable. However, knocking out RSP5 significantly decreased the phosphorylation and activation of the Akt signaling pathway, while overexpressing RSP5 obviously increased the phosphorylation and activation of the Akt signaling pathway. Moreover, the proosteogenic effect of overexpressing RSP5 could be blocked by inhibiting the activation of the Akt signaling pathway. Taken together, these results indicated that RSP5 promoted the osteogenesis of MSCs by activating the Akt signaling pathway. In recent decades, many studies have confirmed that the Akt signaling pathway promotes osteogenesis [7, 13]. The phosphorylation of two key residues on Akt, T308 in the activation domain, or T-loop, of the catalytic protein kinase core and S473 in a C-terminal hydrophobic motif, is required for maximal activation of the kinase [11]. Additionally, Akt is ubiquitylated on multiple distinct Lys residues [11]. Distinct ubiquitin ligases that couple K63-linked ubiquitin to Akt serve to regulate Akt activation. For example, various growth factors elicit activation of the TRAF6 and Skp2 E3 ligases that target Lys residues in the Akt pH domain, and these modifications enhance membrane localization and thus activation [28, 29]. However, most studies only focus on the phosphorylation of Akt during osteogenesis, and few studies have explored the K63-linked ubiquitination of Akt during osteogenic differentiation of MSCs. As mentioned above, RSP5 acts as an E3 ligase to function in most physiological and pathological conditions [9, 30, 31]. For example, Belgareh N demonstrated that RSP5 ubiquitinated ERMES components to control mitophagy [10]. In our study, we demonstrated that RSP5 and Akt interacted with each other and that RSP5 significantly induced the K63-linked ubiquitination of Akt to activate the Akt signaling pathway. To our knowledge, this may be the first study to explore the K63-linked ubiquitination of Akt during osteogenic differentiation of MSCs. Thus, our study may help further expand the network of the Akt signaling pathway.

In conclusion, our study not only expands the knowledge of RSP5 in bone development but also provides a potential target for bone regeneration and repair. However, there are still some limitations of the present study. First, the bone remodeling process is controlled by osteoblasts as well as osteoclasts [32, 33]. Although we demonstrated that RSP5 positively regulates the osteogenic differentiation process of MSCs, the function of RSP5 in osteoclastogenesis has never been explored. Second, although we showed that RSP5 positively regulates the osteogenic differentiation of MSCs in vitro, the concrete role of RSP5 in bone development in vivo still needs further exploration. To overcome the abovementioned limitations, we need to use transgenic mice with specific knockdown or knockdown of RSP5 in osteoblast lineages and osteoclast lineages. Thus, constructing these transgenic mice is our future goal.

## Figures and Tables

**Table 1:** Primer sequences.

Gene	Forward primer (5′-3′)	Reverse primer (5′-3′)
*β-actin*	CATGTACGTTGCTATCCAGGC	CTCCTTAATGTCACGCACGAT
*Runx2*	TCAACGATCTGAGATTTGTGGG	GGGGAGGATTTGTGAAGACGG
*Osteocalcin* (*OCN*)	CACTCCTCGCCCTATTGGC	CCCTCCTGCTTGGACACAAAG
*Osteopontin* (*OPN*)	GAAGTTTCGCAGACCTGACAT	GTATGCACCATTCAACTCCTCG

## Data Availability

All the data used to support the findings of this study are included within the article.

## Supplementary Materials

Supplementary Materials Supplemental Figure 1 (A) Neither the decrease nor overexpression ofRSP5 in MSCs affected the growth curve of these cells in the osteogenic medium. (B) AZD5363 decreased the growth curve of MSCs in the osteogenic induction conditions. ^∗^ indicates *P* < 0.05. *n* = 3 independent experiments with 3 different MSC lines.
