# Corrigendum to “RSP5 Positively Regulates the Osteogenic Differentiation of Mesenchymal Stem Cells by Activating the K63-Linked Ubiquitination of Akt”

**DOI:** 10.1155/2020/4370849

**Published:** 2020-08-21

**Authors:** Changxiang Liang, Guoyan Liang, Xiaoqing Zheng, Yongxiong Huang, Shuaihao Huang, Dong Yin

**Affiliations:** Department of Spinal Surgery, Guangdong Provincial People's Hospital, Guangdong Academy of Medical Sciences, Guangzhou, China 510080

In the article titled “RSP5 Positively Regulates the Osteogenic Differentiation of Mesenchymal Stem Cells by Activating the K63-Linked Ubiquitination of Akt” [1], the affiliation should be changed from “Department of Spinal Surgery, Guangdong Provincial People's Hospital, Guangzhou, China 510080” to the correct affiliation “Department of Spinal Surgery, Guangdong Provincial People's Hospital, Guangdong Academy of Medical Sciences, Guangzhou, China 510080”.

The corrected affiliation is shown in the author information above.
