# Supplementary figures and images for: RSP5 Positively Regulates the Osteogenic Differentiation of Mesenchymal Stem Cells by Activating the K63-Linked Ubiquitination of Akt
Source: Stem Cells Int. 2020 Apr 6;2020:7073805. doi: 10.1155/2020/7073805 (PMC7165343; doi:10.1155/2020/7073805)

**A**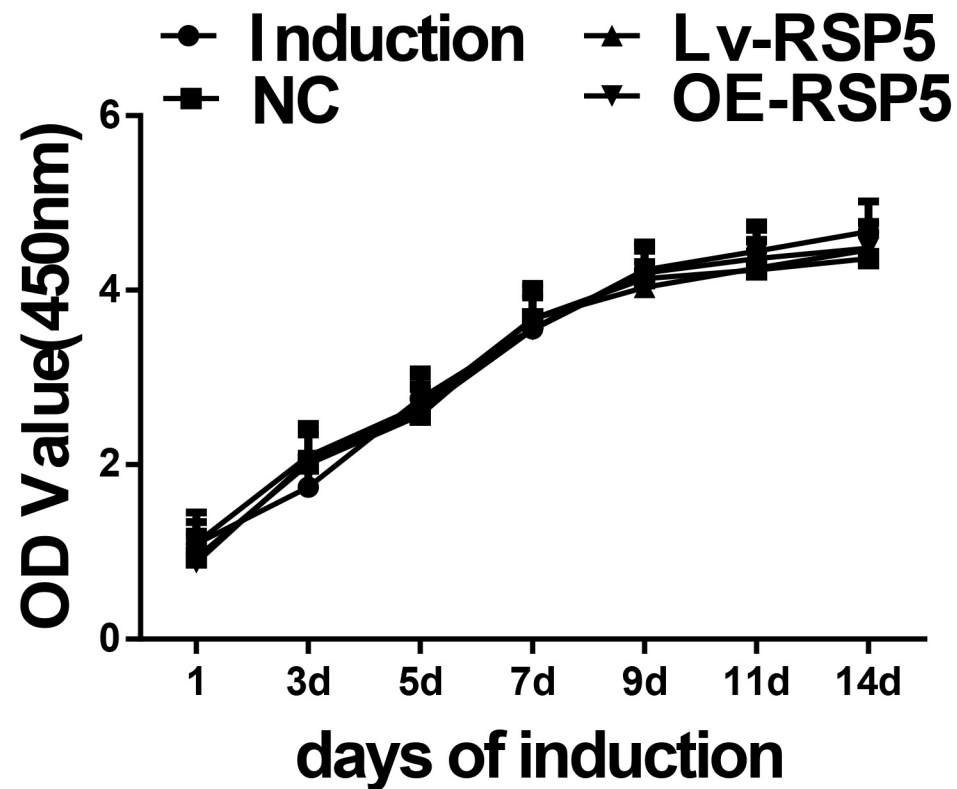**B**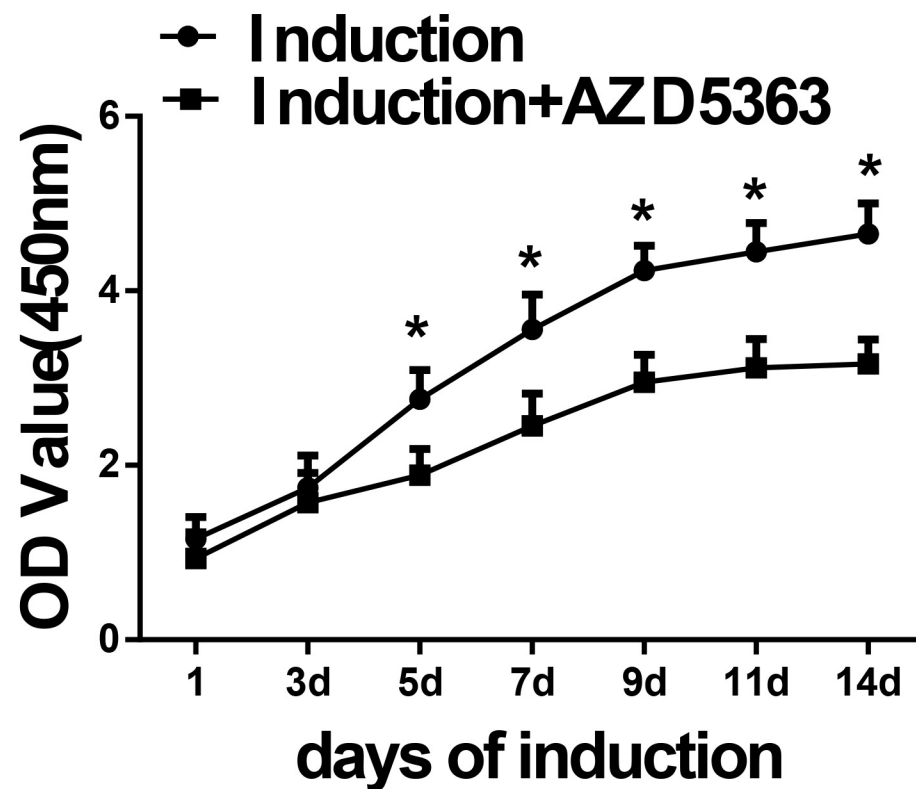

Supplement: Supplementary Materials — Supplemental Figure 1 (A) Neither the decrease nor overexpression ofRSP5 in MSCs affected the growth curve of these cells in the osteogenic medium. (B) AZD5363 decreased the growth curve of MSCs in the osteogenic induction conditions. ∗ indicates P < 0.05. n = 3 independent experiments with 3 different MSC lines. [file 7073805.f1.pdf]
